# COVID-19 prevention behaviors and dietary habits among undergraduate students: A health belief model approach

**DOI:** 10.1371/journal.pone.0309623

**Published:** 2024-08-29

**Authors:** Doreen Liou

**Affiliations:** Department of Nutrition and Food Studies, Montclair State University, Montclair, New Jersey, United States of America; St John’s University, UNITED STATES OF AMERICA

## Abstract

Physical and social disruptions resulted from the COVID-19 pandemic, affecting young adults in higher education. The purpose of this survey research is to unveil COVID-19 related beliefs using the Health Belief Model, and COVID-19 prevention behaviors, including self-reported fruit and vegetable consumption among university students. A cross-sectional survey was administered to 304 male and female undergraduate students from diverse ethnic backgrounds and majors at a New Jersey state university using convenience sampling. Variables measured included frequency of COVID-19 prevention behaviors (e.g.: wearing indoor mask, handwashing), and consumption of fruit and vegetables (dark green and orange vegetables) over the previous week. Health Belief Model constructs were assessed namely perceived susceptibility to COVID-19, severity, benefits, barriers, and self-efficacy. Statistical distributions were computed for the entire sample and t-tests were investigated for subgroups of age and sex. The sample mean age was 21.7 (SD = 4.7) years with 222 females (73%) and 82 males (27%), and 46% identified as White, non-Hispanics. Participants consumed a mean of 0.95 cups of fruit, 0.81 cups of green vegetables, and 0.46 cups of orange vegetables. Female students practiced more COVID-19 prevention behaviors with a mean difference of 0.26 (p<0.001), perceived greater severity from the virus (Δ = 0.37, p = 0.002) and had stronger perceptions of benefits (Δ = 0.18, p = 0.041), barriers (Δ = 0.21, p = 0.046), and self-efficacy (Δ = 0.20, p = 0.020) than their male counterparts. Older students (aged 23 and above) adopted more COVID-19 prevention behaviors (Δ = -0.35, p = 0.001) and perceived less barriers (Δ = 0.24, p = 0.047) than their younger peers. Nutrition educators and health professionals need to emphasize the importance of adopting preventive health behaviors among university students as strategies to mitigate the severity of COVID-19. Addressing barriers younger male and female students may elevate their motivation and self-efficacy to enact health behaviors.

## Introduction

The global pandemic of COVID-19 resulted in colossal physical and social disruptions and impacted the lives of university students in unprecedented ways. In the United States alone, over 100 million COVID-19 cases were reported with common clinical symptoms as fever, weakness, cough, shortness of breath, and in severe cases, pneumonia, and respiratory infections [1, 2]. More than a million fatalities have been documented, especially among individuals with immunocompromised conditions, advanced age, obesity, and other chronic diseases [3]. When individuals are infected with COVID-19, the virus enters human cells and activation of their innate and adaptive immune systems can result in an uptick of pro-inflammatory molecules [4]. Under certain medical conditions, this can cascade to pulmonary injury and respiratory distress. Dysbiosis, or an imbalance in bacterial composition in the gut, may contribute to the severity of COVID-19. Changes in the gut microbiome may play a paramount role in affecting the clinical outcome of COVID-19 [5]. Individuals infected with the virus can undergo a dysregulation of their immune response, however, convalescence can be seen in those with healthy and strong immune systems [6, 7].

Poor diets stemming from excessive intakes of ultra-processed foods, unhealthy fats, and sugars can precipitate physiological stress in the human body. Furthermore, individuals with dysbiosis are more prone to viral infections [8], and nutrient deficiencies (vitamins C, D, and zinc) can weaken cell-mediated immunity [9]. Achieving optimal nutrition is critical for a balanced immune system that acts as a defense mechanism against viral infections. Other factors that can contribute to obesity and deregulate the immune system are inadequate fruit and vegetable consumption along with excess intake of refined carbohydrates, saturated fats, and sedentary lifestyle [10]. During the initial stages of the pandemic, self-isolation and quarantine among young adults promoted sedentary states and unhealthy practices [11]. University students’ adjustments to virtual learning led to many unforeseen physical, social, and emotional challenges such as depressive thoughts, increased levels of stress, and feelings of anxiety and fear [12].

In addressing health concerns, social psychological theories provide a useful framework for comprehending the perceptions and motivations of individuals to adopt behaviors to mitigate risk. The Health Belief Model (HBM) proposes that likelihood of taking a health-related action is motivated by perceptions of risk of contracting a disease or condition (perceived susceptibility) and perceived severity of the condition, which may encompass medical and/or social consequences [13]. Other constructs of the model include the perceived benefits of taking an action to prevent the condition, and the perceived barriers or difficulties of performing that action. These barriers may entail inconvenience, cost, or discomfort. Cues to action are internal events (e.g.: personal symptoms of pain) and external events (such as family illness or news report) that prompt one’s decision to engage in the action. The model was later modified to include self-efficacy or an individual’s confidence to enact a behavior.

Empirical data show support for the application of the HBM with the focus on COVID-19 related beliefs and health behaviors to mitigate its spread among young adults in higher education. Alsulaiman & Rentner [14] used the model to investigate university students’ perceptions of COVID-19 at an American university. Tsai et al. [15] examined nursing students’ relationships between COVID-19 knowledge, beliefs, and behavioral intention to prevent viral spread. The HBM was also used to assess influences and predictors of intention to receive COVID-19 vaccinations among adults [16, 17].

In the United States, an estimated 4 out of 10 American adults (aged 18 to 24) were enrolled in college education, with over 17 million undergraduate students [18]. According to the U.S. Department of Labor, this generation of Gen Z born between 1995 to 2015 constitute over one tenth of the U.S. workforce [19]. Empirical studies point to significant degradation in the dietary habits and nutritional states of college students, marked by low consumption of fruits and vegetables [20, 21] and excessive intakes of fast food. It is paramount for health professionals and educators to understand COVID-19 related beliefs, perceptions, and health behaviors of college students to mitigate viral transmission. The aim of this survey research is to unveil COVID-19 related beliefs using the Health Belief Model and ascertain COVID-19 prevention behaviors, including frequency of fruit and vegetable intake, among undergraduate college students enrolled in a New Jersey state institution. In addition, sub-questions were posed as follows: Are there differences in the behaviors and psychosocial factors measured according to sex and age categories?

## Materials and methods

A convenience sample of college students was recruited at a large northeastern state university in the United States. Eligibility criteria included (a) students enrolled in undergraduate coursework and bachelor’s degrees at a New Jersey state university and (b) male and female commuters and non-commuters representing diverse ethnicities (IRB Number: IRB-FY21-22-2619, Project Title: Survey on COVID-19 Risk and Prevention among Undergraduate Students). Between August 1, 2022, to March 1, 2023, participants were recruited via campus-wide student listservs and academic clubs targeting dietetic students and individuals across different disciplines, ethnicities, and socioeconomic backgrounds. The primary researcher also solicited volunteers at various on-site events and meetings with students from five colleges of the university: College of Education and Human Services, School of Business, College of the Arts, College of Science and Mathematics, and College of Humanities and Social Sciences. Eligible individuals were given information sheets and informed consent forms to complete. Of 434 surveys disseminated, 70% were returned, resulting in a sample size of 304. It was determined that a minimum sample size of 300 is sufficient for this survey research based on the number of study variables and the nature of statistical tests conducted [22]. A sample size calculation for a two-sample t-test to achieve 80% power at α = 0.05 (Cohen’s d and medium effect size) would yield 64 for each sample group (http://www.statskingdom.com). The surveys were completed by respondents between September 2022 to March 2023. On average, participants took approximately 5–10 minutes to complete the 41-item questionnaire. Upon completion of the survey, participants were eligible for a raffle drawing for Amazon gift cards of $25and $50. The anonymity of the participants was ensured by having no identifiable information on the survey. If the respondents entered the raffle drawing, their information was collected on a separate document, and is not connected to their completed survey.

### Measures

Qualitative interviews with undergraduate students at a New Jersey state institution and a comprehensive review of the literature provided a solid contextual framework for the development of the survey instrument [23]. Demographic questions were queried such as age, sex at birth, major of study, race, marital status, birthplace, residence status and campus meal plan.

Two questions were posed on COVID-19 prevention behaviors over the past week, namely, the frequency of wearing a mask indoors when in public and the frequency of handwashing. Respondents indicated on a scale of 1 to 5 with ‘1’ denoting never and ‘5’ representing always. Participants’ consumption of fruits and dark green and orange vegetables over the previous week were measured by the number of cups consumed from Monday to Sunday.

A total of six variables derived from the HBM addressed participants’ perceptions of susceptibility and severity to COVID-19, benefits of diet-related actions, perceived barriers, cues to action, and self-efficacy to enact health behaviors. Respondents indicated on a scale of 1 to 5 the degree to which they agreed or disagreed with each statement (‘1’ denoted ‘strongly disagree’ and ‘5’ as ‘strongly agree’). Sample survey items are displayed in Table 1.

**Table 1:** Examples of questionnaire items.

Constructs	Sample Questionnaire Statements
**Health Behaviors**	
COVID-19 Prevention Behaviors	Over the past week, how often did you wear a mask indoors when in public?
	Over the past week, how often did you practice handwashing?
Fruit Consumption	Over the past week, approximately how many cups of fruit did you consume on each day?
Vegetable Consumption	Over the past week, approximately how many cups of dark green vegetables did you consume each day?
	Over the past week, approximately how many cups of orange vegetables (e.g.: carrots, squash, sweet potato) did you consume on each day?
**Health Belief Model**	
Perceived Susceptibility	I am very likely to contract COVID-19 or other coronaviruses in the upcoming month.
Perceived Severity	I will experience negative mental effects (e.g.: depression, anxiety) if I contract COVID-19.
Perceived Benefits	Wearing a mask in public will lessen my chances of contracting COVID-19.
Perceived Barriers	Eating healthfully will be costly for me.
Cues to Action	When I do not feel well, I am prompted to eat more fruits and vegetables.
Self-Efficacy	I am confident in my ability to select foods that are beneficial to my immune system.

### Questionnaire validity and reliability

Face validity was assessed via a separate pilot sample of 20 students who provided feedback about the clarity and meaning of the questionnaire items. Nutrition and behavior science faculty reviewed the instrument for content validity and accurate reflection of the Health Belief Model from literature review. Reliability was measured using Cronbach’s alpha internal consistency assessment. The Cronbach’s alpha (goodness of fit) calculation was employed to examine the reliability of the instrument. The Cronbach’s alpha coefficients on the six Health Belief Model constructs were above 0.70, reflecting acceptable internal consistency.

Exploratory factor analyses were employed to examine construct validity. The extraction method used was principal component analysis and the rotation method was direct oblimin with Kaiser normalization. Bartlett’s Test of Sphericity indicated that the correlation matrix has significant correlations among select variables in the dataset (p<0.001). Factor analyses of the HBM variables produced 7 distinct factors, accounting for 59% of the variance in responses. The marker indicator used in the confirmatory factor analysis model is factor loading. After additional factor analysis for each subscale, 2 items had factor loadings of less than 0.40 and were deleted from the scale. The rest of the factor loadings were >0.4, revealing tight relations between factors and items. The Kaiser-Meyer-Olkin Measure of Sampling Adequacy was 0.81, indicating that the distribution of values is strong for conducting factor analysis, further supporting a reasonable model-data fit.

To establish relative validity, dietary questions on daily fruit and vegetables (green and orange) over the previous week were validated against 3-day food records of 20 undergraduate students enrolled in an introductory nutrition course at the university. A Pearson correlation of 0.61 was statistically significant for fruit intake (p<0.01). For both green and orange vegetables, a Pearson correlation of approximately 0.20 was recorded (p = 0.53). Potential reasons for the low correlation observed for vegetable intake include a small sample size of undergraduates and lack of accessibility of vegetables during the COVID-19 pandemic.

### Data analysis

Quantitative analysis was employed using the IBM Statistical Package for the Social Sciences (SPSS) version 23.0. The data analysis included frequency distributions, calculations for central tendency and dispersion for the behavioral, psychosocial, and demographic variables. Cronbach’s alpha coefficients were calculated for reliability testing of the psychosocial constructs and confirmatory factor analyses were conducted to establish construct validity. Independent t-tests were performed to assess for differences in mean values for the behavioral and psychosocial factors based on subgroups for age and sex. Participants’ age was further categorized as ‘younger’ (between 18 to 22 years) and ‘older’ (23 years and above), based on the university’s demographic profile for undergraduate students.

## Results

### Demographic characteristics

The mean age of the sample was 21.7 ± 4.7 with 73% females and 46% identified as White, non-Hispanics. Based on age categories, approximately 80% of the sample were categorized as ‘younger’ or between the ages of 18 to 22. The participants were primarily single or never married (93%) with 34% declaring health sciences (e.g.: nutrition, biology, exercise science, public health) as their major of study at the university. Approximately 73% of the respondents were commuters, while 27% of the students had dormitory residences (Table 2).

**Table 2:** Demographic data of university students.

Demographic Factor	n	%
**Sex:**		
Male	82	27.0
Female	222	73.0
**Age Group:**		
Younger Group (18–22 years)	244	80.3
Older Group (23 years and above)	60	19.7
Mean Age (SD) = 21.7 (4.7)		
**Race:**		
White, non-Hispanic	139	45.7
Black	46	15.1
Hispanic/Latino	80	26.3
Asian Pacific Islander	26	8.6
Two or More Races	13	4.3
**Academic Major:**		
Health Science	103	34.2
Non-Health	198	65.1
Undeclared	3	0.7
**Marital Status:**		
Single or Never Married	284	93.4
Married	19	6.3
Divorced	1	0.3
**Residence Status:**		
Campus Dormitory	81	26.6
Commuter	223	73.4

### COVID-19 prevention behaviors and fruit/vegetable consumption

For the entire sample, participants’ mean score for practicing COVID-19 prevention behaviors was 3.13 ± 0.61, reflecting ‘sometimes’ in wearing indoor masks and practicing handwashing. Over the previous week, respondents consumed approximately 0.95 ± 1.00 cups of fruit per day, 0.81 ± 0.83 cups of green vegetables and 0.46 ± 0.60 cups of orange vegetables per day.

### Perceived susceptibility & severity

When questioned about their susceptibility of contracting COVID-19, a mean score of 2.33 ± 0.79 (range of 1 to 5) reflected disagreement in their likelihood of contracting the virus. Survey items addressed one’s chances of contracting the virus due to a compromised immune system, taking classes in-person, and having active social interactions in public.

Participants were surveyed about the physical, mental, and social consequences of contracting COVID-19. The mean score of perceived severity was 2.90 ± 0.89 (range of 1 to 5).

Perceived severity measured notable physical effects such as fever, fatigue, cough, and being contagious to high-risk population groups. Negative mental effects of having COVID-19 included depression, anxiety, and social isolation.

### Perceived benefits

For the entire sample, the mean value for perceived benefits was 3.83 ± 0.66, reflecting a general agreement that health behaviors can reduce one’s risk of contracting COVID-19. These health actions entailed consuming lots of fruits and vegetables, wearing a mask in public, consuming vitamins C and D via food or supplements, handwashing, and getting vaccinated and/or boosted.

### Perceived barriers

For the entire sample, the mean value for perceived barriers was 3.01 ± 0.66, reflecting the degree in which obstacles such as accessibility of plant-based foods, lack of time to prepare home meals, cost, academic stress and the negative effects of social media impacted the respondents.

### Self-efficacy

The mean value for self-efficacy was 3.81 ± 0.65, reflecting the sample’s relative confidence in adopting health behaviors. The four items measuring self-efficacy encompassed confidence in preparing nutritionally balanced meals, viewing social media posts to enhance healthy cooking, selecting foods to benefit the immune system, and ability to update COVID-19 vaccination status.

### Cues to action

Lastly, assessment of cues to action resulted in a mean value of 3.07 ± 0.86 for the entire sample. Five items measured cues to consume more vitamin C in one’s diet due to upticks in COVID-19 cases, maintaining social distance when someone coughs in an indoor public setting, wearing indoor masks due to hearing or reading about Centers for Disease Control and Prevention (CDC) recommendations, and enrolling in available online courses due to concerns over contagious viruses.

### Comparisons of COVID-19 preventive behaviors and health beliefs by sex

Female participants practiced more COVID-19 prevention behaviors (p<0.001), perceived greater threat from the virus (p<0.01) and had stronger perceptions of benefits, barriers, and self-efficacy (p<0.05) than their male counterparts (Table 3).

**Table 3:** Comparisons on behavior and health belief model constructs according to sex using independent t-test.

Category	Male Mean (SD)	Female Mean (SD)	Mean Difference	95% CI	Sig. (2-tailed)
**Health Behaviors**					
COVID-19 Prevention (range 1 to 5)	2.94 (0.52)	3.20 (0.62)	0.26	0.11, 0.41	p<0.001***
Fruit Consumption (cups)	0.93 (0.80)	0.95 (1.06)	0.02	-0.23, 0.28	p = 0.869
Vegetable Consumption (cups)	0.53 (0.47)	0.67 (0.70)	0.14	-0.02, 0.31	p = 0.088
**Health Belief Model (range 1 to 5)**					
Perceived Susceptibility	2.07 (0.77)	2.42 (0.78)	0.35	0.15, 0.55	p<0.001***
Perceived Severity	2.63 (0.86)	3.00 (0.89)	0.37	0.14, 0.59	p = 0.002**
Perceived Benefits	3.70 (0.73)	3.87 (0.63)	0.18	0.01, 0.34	p = 0.041*
Perceived Barriers	2.86 (0.78)	3.07 (0.82)	0.21	0.00, 0.42	p = 0.046*
Cues to Action	2.71 (0.89)	3.20 (0.81)	0.49	0.28, 0.70	p<0.001***
Self-Efficacy	3.67 (0.61)	3.86 (0.65)	0.20	0.03, 0.36	p = 0.020*

* p<0.05

** p<0.01

*** p<0.001

### Comparisons of COVID-19 preventive behaviors and health beliefs by age group

Older students (ages 23 and above) adopted more COVID-19 prevention behaviors (p<0.01) and consumed more vegetables (p<0.05) than their younger peers (ages 18–22), while younger students perceived greater barriers (Table 4).

**Table 4:** Comparisons on behavior and health belief model constructs according to age group using independent t-test.

Category	Younger Group Mean (SD)	Older Group Mean (SD)	Mean Difference	95% CI	Sig. (2-tailed)
**Health Behaviors**					
COVID-19 Prevention (range 1 to 5)	3.06 (0.55)	3.41 (0.73)	-0.35	-0.56, -0.14	p = 0.001**
Fruit Consumption (cups)	0.91 (0.91)	1.13 (1.31)	-0.23	-0.60, 0.14	p = 0.129
Vegetable Consumption (cups)	0.60 (0.54)	0.78 (0.96)	-0.18	-0.37, 0.01	p = 0.183
**Health Belief Model** (range 1 to 5)					
Perceived Susceptibility	2.31 (0.80)	2.42 (0.72)	-0.11	-0.34, 0.13	p = 0.369
Perceived Severity	2.91 (0.88)	2.94 (0.88)	-0.03	-0.28, 0.23	p = 0.839
Perceived Benefits	3.80 (0.66)	3.88 (0.65)	-0.08	-0.27, 0.12	p = 0.435
Perceived Barriers	3.06 (0.80)	2.82 (0.80)	0.24	0.00, 0.47	p = 0.047*
Cues to Action	3.03 (0.87)	3.24 (0.83)	-0.21	-0.46, 0.04	p = 0.099
Self-Efficacy	3.77 (0.65)	3.94 (0.65)	-0.17	-0.36, 0.02	p = 0.084

* p<0.05

** p<0.01

## Discussion

In this sample, self-reported intake of fruit and vegetables fell below the U.S. federal recommendations for a healthy eating pattern, which are that adults consume at least 1.5–2 cups of fruit per day and 2–3 cups of vegetables per day. According to a published study, only 9% of adults met the intake recommendations for vegetables and 12% of adults met the recommendations for fruit [24]. Furthermore, Lee-Kwan et al. revealed that lower consumption of fruit and vegetables was more pronounced in men and young adults between the ages of 18 to 30. The Centers of Disease Prevention and Control have found that high cost, lack of preparation time, and limited access and availability are obstacles to the consumption of these plant-based foods. Barriers to the intake of balanced, nutrient-dense diets among university students included the plethora of fast food and junk foods surrounding their campus and communities [23]. Lack of time due to course-related assignments during the academic year also predisposed individuals to poor food choices and inability to cook their own meals. Financial concerns impacted the food purchasing decisions of this sample of college students. Limited discretionary spending money due to the rising costs of paying for a college education may predispose individuals to purchase cheap, fast-food items high in salt, unhealthy fats, and sugars.

In relation to the HBM, Alsulaiman & Rentner [14] found a significant relationship between perceived threat of COVID-19 and likelihood to wear a mask in public settings among American college students. Furthermore, Qiao et al. [25] reported that students’ perceived severity of COVID-19 was positively associated with vaccine acceptance. Some other beliefs that were strong predictors of intention to receive the COVID-19 vaccine included positive attitudes towards vaccination, lower concerns related to vaccine safety, and consulting social media as a source of information [26]. A high level of digital health literacy also impacted vaccine acceptance in addition to belief of COVID-19 susceptibility and a belief that infection would severely impact one’s life [27].

Significant positive correlations between fear of COVID-19 and depression and anxiety were detected in a study by Ahorsu et al. [28] Taylor [29] referred to psychological vulnerability factors as playing a part in the fear of COVID-19, including intolerance of uncertainty and perceived susceptibility to disease and anxiety proneness. When evaluating overall preventative behaviors such as wearing a mask in public, handwashing, and proper diet and exercise, there were multiple factors that contributed to students’ willingness to participate in these behaviors. The perceived threat of COVID-19 had a significant correlation with the adoption of preventative behaviors [14]. Risk perception is also shown to affect the practice of preventive behaviors in studies done in Iran and Italy where students with high performance in preventative behaviors showed moderate and high-risk perception respectively [30, 31] Shumway et al. [32] examined predictors of compliance with COVID-19 related non-pharmacological interventions (e.g.: wearing masks, social distancing) among university students in the U.S. Concern about the severity of infections and news exposure had a significant positive correlation with compliance among the participants.

This New Jersey study sample perceived benefits of enacting behaviors to reduce risk of COVID-19 infection. Kim et al. [21] applied the HBM to assess how university students’ nutrition beliefs influence intention to adopt health behaviors. Students’ perceived benefits of healthy eating had significant effects on their intentions. In another study utilizing the HBM, perceived benefits were influential in developing intention to get vaccinated [16] Another study reported that higher perceived benefits and higher perceived susceptibility to COVID-19 was found to be salient predictors for taking the vaccines [17].

This New Jersey sample revealed that females and younger undergraduates generally expressed stronger barriers in practicing health behaviors. Based on Powell et al.’s study [33], displacement from on campus housing during the pandemic affected student food choices. Some students who had a food plan on campus but returned to family homes where their parents were the primary shoppers reported eating food significantly lower in nutrient content and caloric value. Students reported limited availability of fresh produce, purchasing more non-perishable foods during the pandemic. They also reported additional free time which contributed to increased snacking behavior. This can also be related to why food insecure students significantly decreased their fruit and vegetable intake during the pandemic [34].

In terms of self-efficacy, this New Jersey sample demonstrated that females and older university students expressed stronger confidence to enact health behaviors. Alsulaiman & Rentner [35] reported that college students with higher scores of self-efficacy were more likely to adopt preventive measures to lower their risk of contracting Middle East Respiratory Syndrome Coronavirus (MERS-CoV). Factors that would increase young adults’ confidence to engage in dietary practices were solicited by Liou & Karasik’s [23] qualitative study. Among GenZers, nutritional knowledge can be obtained via Google searches, YouTube videos, and social media platforms such as Instagram, TikTok, and Facebook. College students often view fitness and culinary influencers as role models. Self-efficacy can be enhanced by social norms entailing peer support for healthy eating and collective influence in consuming fruit and vegetables at home and on campus.

Many studies showed an overall change in health and nutrition behaviors in undergraduate students during lockdown [33, 36] although study results can be affected by external circumstances of the students and their intrapersonal approaches to eating. One study showed that students who were categorized with high scores on the Satter Eating Competence (EC) Inventory reported eating more fruits and vegetables and more home-cooked meals during the pandemic than non-EC students [37]. Non-EC individuals reported negative changes in their diet such as eating more takeout, processed foods and more sugary items.

Another study done in Italy found that students who were not enrolled in a life sciences major also improved their diet while life sciences majors made no change [31]. This could be because the life sciences students already had the knowledge and ability to maintain a healthy diet before the pandemic, thus not needing to make changes to their diet. The other students, however, needed to make changes in response to the risk of infection. Life science students displayed greater awareness towards COVID-19 infection mechanism and ways to mitigate its spread. In a study conducted in China, students reported an increase in nutrition behavior, health responsibility, and social support when compared to before the pandemic [38].

Lastly, one study done in Germany indicated increased food intake during the pandemic lockdown was more likely in female participants, students with higher mental stress, and those with higher body mass indexes [36]. Kim et al. [39] identified factors affecting the practice of COVID-19 prevention behaviors among college students in Korea. Knowledge attainment from education on infectious disease was shown to be highly related to adoption of health behaviors. Female students expressed more positive attitudes toward the health behaviors than their male counterparts.

## Conclusions

The COVID-19 pandemic impacted the perceptions and behavioral practices of American university students. This survey research highlighted empirical data on the beliefs of COVID-19 susceptibility, its severity, benefits of health actions to reduce risk, perceived barriers, and confidence to enact health behaviors. These key psychosocial underpinnings from the Health Belief Model provide valuable insights for health professionals, researchers, and educators serving this growing population of young adults in the United States. In this sample of 304 individuals, females practiced more health actions and perceived a greater threat from the virus. Furthermore, they expressed higher levels of benefits, barriers, and self-efficacy than their male counterparts. Older students performed more COVID-19 prevention behaviors and consumed more vegetables than their younger constituents.

In this study, self-reported consumption of fruit and vegetables revealed inadequate amounts as compared to U.S. federal guidelines for a healthy dietary pattern to reduce risk of illness and chronic diseases. A healthy immune system is vital to mitigate viral infections due to COVID-19. Prioritizing efforts to identify and address barriers to fruit and vegetable consumption among young adults is necessary.

This study offers important insights on the psychosocial beliefs and behavioral practices of young adults, using the Health Belief Model as a guiding theoretical framework. The strengths of this research are the validation and reliability of a survey instrument designed to assess COVID-19 prevention behaviors, perceptions of threat, benefits and barriers of enacting health actions, and self-efficacy. Several caveats existed reflecting a relatively small sample size from a single university in the U.S. However, the researcher aimed to recruit and survey individuals from diverse academic majors, years of study, and ethnicities across campus to secure a near representative sample. In this study sample, a greater portion of female students and participants from a younger age group could potentially affect study outcomes. Secondly, generalizability of findings to all undergraduate students in the U.S. is limited with a convenience sample since a random sample was not applied. Random sampling was not applied due to logistical challenges with university policies. Also, many departmental student listservs are voluntary and not all students opt-in. Thirdly, survey data was collected after American universities reopened for in-person classes and mask mandates were lifted. This period of data collection may impact respondents’ beliefs and practices reflecting indoor mask-wearing behavior.

Future research is warranted to unveil COVID-19 related beliefs and prevention behaviors of college students in other geographic regions in the country (e.g.: urban, suburban) and from varying socioeconomic backgrounds. Using the Health Belief Model as a guiding theoretical framework, additional work is necessitated on deeply probing the relationships between perceived threat and ways to increase self-efficacy to execute health behaviors across age and gender subgroups. Perceptions of viral risk and the benefits and barriers to the adoption of health behaviors need to be identified for effective campaigns and communication strategies to be developed. Findings from this quantitative study can fortify university educators and nutritionists with the insights needed to promote the consumption of fruit and vegetables for optimal health to defend against new strains of COVID-19.

## Supporting information

S1 Appendix Frequency analyses. (PDF)
