# Supplementary material for: COVID-19 prevention behaviors and dietary habits among undergraduate students: A health belief model approach
Source: PLoS One. 2024 Aug 29;19(8):e0309623. doi: 10.1371/journal.pone.0309623 (PMC11361559; doi:10.1371/journal.pone.0309623)
Supplement: S1 Appendix — (PDF) [file pone.0309623.s001.pdf]

```
FREQUENCIES VARIABLES=E2
 /STATISTICS=STDDEV VARIANCE RANGE MEAN MEDIAN MODE
 /ORDER=ANALYSIS.
```

Frequencies

Statistics

Age

N	Valid	301
	Missing	3
Mean		21.7209
Median		20.0000
Mode		20.00
Std. Deviation		4.74783
Variance		22.542
Range		32.00

Age

		Frequency	Percent	Valid Percent	Cumulative Percent
Valid	17.00	1	.3	.3	.3
	18.00	29	9.5	9.6	10.0
	19.00	47	15.5	15.6	25.6
	20.00	77	25.3	25.6	51.2
	21.00	56	18.4	18.6	69.8
	22.00	34	11.2	11.3	81.1
	23.00	15	4.9	5.0	86.0
	24.00	7	2.3	2.3	88.4
	25.00	5	1.6	1.7	90.0
	26.00	3	1.0	1.0	91.0
	27.00	2	.7	.7	91.7
	28.00	4	1.3	1.3	93.0
	29.00	1	.3	.3	93.4
	30.00	5	1.6	1.7	95.0
	31.00	3	1.0	1.0	96.0
	32.00	1	.3	.3	96.3
	33.00	2	.7	.7	97.0
	37.00	1	.3	.3	97.3

Age

		Frequency	Percent	Valid Percent	Cumulative Percent
	40.00	1	.3	.3	97.7
	41.00	2	.7	.7	98.3
	43.00	1	.3	.3	98.7
	44.00	1	.3	.3	99.0
	47.00	1	.3	.3	99.3
	48.00	1	.3	.3	99.7
	49.00	1	.3	.3	100.0
	Total	301	99.0	100.0	
Missing	System	3	1.0		
Total		304	100.0		

```
FREQUENCIES VARIABLES=E2 A1 A2 E3 E4 E5Race E6 FRUIT VEGGREEN VEGORANGE SUSCEPT SEVERITY
BENEFITS
    SE BARRIERS CUES BCOVID VEGETABLES
  /STATISTICS=STDDEV VARIANCE RANGE MEAN MEDIAN MODE
  /ORDER=ANALYSIS.
```

Frequencies

Notes

Output Created		10-MAR-2024 16:28:22
Comments		
Input	Data	C:\Users\lioud\Documents\LIOU SURVEY COVID 2023 Sept 11.sav
	Active Dataset	DataSet1
	Filter	<none>
	Weight	<none>
	Split File	<none>
	N of Rows in Working Data File	304
Missing Value Handling	Definition of Missing	User-defined missing values are treated as missing.
	Cases Used	Statistics are based on all cases with valid data.
Syntax		FREQUENCIES VARIABLES=E2 A1 A2 E3 E4 E5Race E6 FRUIT VEGGREEN VEGORANGE SUSCEPT SEVERITY BENEFITS SE BARRIERS CUES BCOVID VEGETABLES /STATISTICS=STDDEV VARIANCE RANGE MEAN MEDIAN MODE /ORDER=ANALYSIS.
Resources	Processor Time	00:00:00.00
	Elapsed Time	00:00:00.01

Statistics

		Age	Mask Freq	Handwashing Freq	Sex	Major	Race
N	Valid	301	304	304	304	304	304
	Missing	3	0	0	0	0	0
Mean		21.7209	1.5658	4.7007	1.2697		4.6842
Median		20.0000	1.0000	5.0000	1.0000		4.0000
Mode		20.00	1.00	5.00	1.00		6.00
Std. Deviation		4.74783	.98618	.67916	.45192		1.50432
Variance		22.542	.973	.461	.204		2.263
Range		32.00	4.00	4.00	2.00		6.00

Statistics

		Marital Status	FRUIT	VEGGREEN	VEGORANGE	SUSCEPT	SEVERITY
N	Valid	304	304	304	304	304	304
	Missing	0	0	0	0	0	0
Mean		3.8092	.9458	.8112	.4580	2.3265	2.8997
Median		4.0000	.7143	.6821	.2857	2.2500	3.0000
Mode		4.00	1.00	.00	.00	2.50	3.25
Std. Deviation		.72879	.99518	.82645	.59895	.79194	.89289
Variance		.531	.990	.683	.359	.627	.797
Range		3.00	8.00	8.00	5.71	3.50	4.00

Statistics

		BENEFITS	SE	BARRIERS	CUES	BCOVID	VEGETABLES
N	Valid	304	304	304	304	304	304
	Missing	0	0	0	0	0	0
Mean		3.8268	3.8076	3.0125	3.0724	3.1332	.6346
Median		3.8333	4.0000	3.0000	3.2000	3.0000	.5000
Mode		3.83	4.00	3.20	3.40	3.00	.00
Std. Deviation		.65861	.64994	.81145	.85644	.61164	.64627
Variance		.434	.422	.658	.733	.374	.418
Range		4.00	3.50	4.00	4.00	4.00	6.86

Frequency Table

Mask Freq

		Frequency	Percent	Valid Percent	Cumulative Percent
Valid	Never	208	68.4	68.4	68.4
	Rarely	47	15.5	15.5	83.9
	Sometimes	29	9.5	9.5	93.4
	Often	13	4.3	4.3	97.7
	Always	7	2.3	2.3	100.0
	Total	304	100.0	100.0	

Handwashing Freq

		Frequency	Percent	Valid Percent	Cumulative Percent
Valid	Never	3	1.0	1.0	1.0
	Rarely	3	1.0	1.0	2.0
	Sometimes	11	3.6	3.6	5.6
	Often	48	15.8	15.8	21.4
	Always	239	78.6	78.6	100.0
	Total	304	100.0	100.0	

Sex

		Frequency	Percent	Valid Percent	Cumulative Percent
Valid	Female	223	73.4	73.4	73.4
	Male	80	26.3	26.3	99.7
	3.00	1	.3	.3	100.0
	Total	304	100.0	100.0	

Major

		Frequency	Percent	Valid Percent	Cumulative Percent
Valid		1	.3	.3	.3
	Account	1	.3	.3	.7
	Accounti	2	.7	.7	1.3
	Advertis	2	.7	.7	2.0
	Arts	1	.3	.3	2.3
	Athletic	4	1.3	1.3	3.6
	Biology	2	.7	.7	4.3
	Business	16	5.3	5.3	9.5
	C. Justi	1	.3	.3	9.9
	Child Ad	2	.7	.7	10.5
	Communi	2	.7	.7	11.2
	Communic	4	1.3	1.3	12.5
	Computer	3	1.0	1.0	13.5
	Criminal	2	.7	.7	14.1
	Dance	1	.3	.3	14.5
	Data Sci	1	.3	.3	14.8
	Dietetic	2	.7	.7	15.5
	Econom	1	.3	.3	15.8
	Economic	2	.7	.7	16.4
	Educ Fou	2	.7	.7	17.1
	English	1	.3	.3	17.4
	Ex. Scie	1	.3	.3	17.8
	Exc Sci	1	.3	.3	18.1
	Excer Sc	1	.3	.3	18.4
	Exer Sci	9	3.0	3.0	21.4
	exercise	1	.3	.3	21.7
	Fam Sci	30	9.9	9.9	31.6
	Family	3	1.0	1.0	32.6
	Fashion	21	6.9	6.9	39.5
	Film TV	3	1.0	1.0	40.5
	Food Sci	1	.3	.3	40.8
	Food sys	1	.3	.3	41.1
	Food Sys	1	.3	.3	41.4
	FSHD	1	.3	.3	41.8
	History	2	.7	.7	42.4

Major

	Frequency	Percent	Valid Percent	Cumulative Percent
Hospita	1	.3	.3	42.8
Hospital	37	12.2	12.2	54.9
HSET	1	.3	.3	55.3
InfoTech	1	.3	.3	55.6
Justice	4	1.3	1.3	56.9
Linguist	2	.7	.7	57.6
Marketin	1	.3	.3	57.9
Math	2	.7	.7	58.6
Musical	1	.3	.3	58.9
Nursing	3	1.0	1.0	59.9
Nutition	1	.3	.3	60.2
Nutritio	63	20.7	20.7	80.9
Nutriton	1	.3	.3	81.3
Nutrtion	1	.3	.3	81.6
Paralega	1	.3	.3	81.9
Phy Educ	1	.3	.3	82.2
Phys Ed	1	.3	.3	82.6
Politica	5	1.6	1.6	84.2
Psych	11	3.6	3.6	87.8
Psycho	4	1.3	1.3	89.1
Psychol	1	.3	.3	89.5
Psycholo	3	1.0	1.0	90.5
Public h	1	.3	.3	90.8
Public H	3	1.0	1.0	91.8
Public R	1	.3	.3	92.1
Religion	1	.3	.3	92.4
Sociolog	2	.7	.7	93.1
SportMgt	1	.3	.3	93.4
Sports	3	1.0	1.0	94.4
Sports C	1	.3	.3	94.7
Sports M	1	.3	.3	95.1
Theatre	1	.3	.3	95.4
TV Media	1	.3	.3	95.7
Undecid	2	.7	.7	96.4
Undecide	7	2.3	2.3	98.7

Major

		Frequency	Percent	Valid Percent	Cumulative Percent
	Undeclar	1	.3	.3	99.0
	Visual	1	.3	.3	99.3
	Visual A	2	.7	.7	100.0
	Total	304	100.0	100.0	

Race

		Frequency	Percent	Valid Percent	Cumulative Percent
Valid	American Indian Alaskan	2	.7	.7	.7
	Asian	26	8.6	8.6	9.2
	Black Aftrican American	46	15.1	15.1	24.3
	Hispanic Latino	80	26.3	26.3	50.7
	White Caucasian	138	45.4	45.4	96.1
	Two or more races	12	3.9	3.9	100.0
	Total	304	100.0	100.0	

Marital Status

		Frequency	Percent	Valid Percent	Cumulative Percent
Valid	Married	19	6.3	6.3	6.3
	Divorced	1	.3	.3	6.6
	Single or Never Married	284	93.4	93.4	100.0
	Total	304	100.0	100.0	

FRUIT

		Frequency	Percent	Valid Percent	Cumulative Percent
Valid	.00	33	10.9	10.9	10.9
	.07	1	.3	.3	11.2
	.14	16	5.3	5.3	16.4
	.27	1	.3	.3	16.8
	.29	25	8.2	8.2	25.0
	.30	3	1.0	1.0	26.0
	.36	2	.7	.7	26.6
	.43	30	9.9	9.9	36.5
	.50	1	.3	.3	36.8
	.56	1	.3	.3	37.2
	.57	19	6.3	6.3	43.4
	.64	2	.7	.7	44.1
	.70	1	.3	.3	44.4
	.71	24	7.9	7.9	52.3
	.86	14	4.6	4.6	56.9
	1.00	52	17.1	17.1	74.0
	1.14	5	1.6	1.6	75.7
	1.18	1	.3	.3	76.0
	1.29	11	3.6	3.6	79.6
	1.36	2	.7	.7	80.3
	1.43	6	2.0	2.0	82.2
	1.50	1	.3	.3	82.6
	1.57	10	3.3	3.3	85.9
	1.71	4	1.3	1.3	87.2
	1.86	3	1.0	1.0	88.2
	2.00	13	4.3	4.3	92.4
	2.14	3	1.0	1.0	93.4
	2.29	1	.3	.3	93.8
	2.43	1	.3	.3	94.1
	2.57	1	.3	.3	94.4
	2.71	3	1.0	1.0	95.4
	3.00	5	1.6	1.6	97.0
	3.29	2	.7	.7	97.7
	3.43	1	.3	.3	98.0
	3.86	1	.3	.3	98.4

FRUIT

		Frequency	Percent	Valid Percent	Cumulative Percent
	4.00	2	.7	.7	99.0
	5.57	1	.3	.3	99.3
	8.00	2	.7	.7	100.0
	Total	304	100.0	100.0	

VEGGREEN

		Frequency	Percent	Valid Percent	Cumulative Percent
Valid	.00	51	16.8	16.8	16.8
	.04	1	.3	.3	17.1
	.07	2	.7	.7	17.8
	.09	1	.3	.3	18.1
	.14	20	6.6	6.6	24.7
	.29	25	8.2	8.2	32.9
	.30	2	.7	.7	33.6
	.36	2	.7	.7	34.2
	.37	1	.3	.3	34.5
	.43	18	5.9	5.9	40.5
	.44	1	.3	.3	40.8
	.50	1	.3	.3	41.1
	.57	24	7.9	7.9	49.0
	.60	1	.3	.3	49.3
	.63	1	.3	.3	49.7
	.68	1	.3	.3	50.0
	.69	1	.3	.3	50.3
	.71	23	7.6	7.6	57.9
	.79	1	.3	.3	58.2
	.86	15	4.9	4.9	63.2
	.93	1	.3	.3	63.5
	1.00	34	11.2	11.2	74.7
	1.07	1	.3	.3	75.0
	1.14	8	2.6	2.6	77.6
	1.21	1	.3	.3	78.0
	1.29	12	3.9	3.9	81.9
	1.43	4	1.3	1.3	83.2

VEGGREEN

	Frequency	Percent	Valid Percent	Cumulative Percent
1.50	1	.3	.3	83.6
1.57	6	2.0	2.0	85.5
1.71	4	1.3	1.3	86.8
1.86	6	2.0	2.0	88.8
2.00	16	5.3	5.3	94.1
2.11	1	.3	.3	94.4
2.14	3	1.0	1.0	95.4
2.29	5	1.6	1.6	97.0
2.43	1	.3	.3	97.4
2.50	1	.3	.3	97.7
2.57	2	.7	.7	98.4
2.71	1	.3	.3	98.7
3.00	1	.3	.3	99.0
3.43	1	.3	.3	99.3
4.00	1	.3	.3	99.7
8.00	1	.3	.3	100.0
Total	304	100.0	100.0	

VEGORANGE

		Frequency	Percent	Valid Percent	Cumulative Percent
Valid	.00	102	33.6	33.6	33.6
	.04	1	.3	.3	33.9
	.07	3	1.0	1.0	34.9
	.09	2	.7	.7	35.5
	.14	26	8.6	8.6	44.1
	.19	1	.3	.3	44.4
	.21	1	.3	.3	44.7
	.27	1	.3	.3	45.1
	.27	1	.3	.3	45.4
	.29	32	10.5	10.5	55.9
	.30	1	.3	.3	56.3
	.32	1	.3	.3	56.6
	.43	26	8.6	8.6	65.1
	.57	19	6.3	6.3	71.4
	.71	18	5.9	5.9	77.3
	.79	1	.3	.3	77.6
	.86	8	2.6	2.6	80.3
	.93	1	.3	.3	80.6
	1.00	28	9.2	9.2	89.8
	1.14	4	1.3	1.3	91.1
	1.29	12	3.9	3.9	95.1
	1.43	2	.7	.7	95.7
	1.57	2	.7	.7	96.4
	1.71	3	1.0	1.0	97.4
	2.00	2	.7	.7	98.0
	2.14	1	.3	.3	98.4
	2.26	1	.3	.3	98.7
	2.29	1	.3	.3	99.0
	2.43	1	.3	.3	99.3
	3.00	1	.3	.3	99.7
	5.71	1	.3	.3	100.0
	Total	304	100.0	100.0	

SUSCEPT

		Frequency	Percent	Valid Percent	Cumulative Percent
Valid	1.00	33	10.9	10.9	10.9
	1.25	15	4.9	4.9	15.8
	1.50	16	5.3	5.3	21.1
	1.75	20	6.6	6.6	27.6
	2.00	35	11.5	11.5	39.1
	2.25	36	11.8	11.8	51.0
	2.50	40	13.2	13.2	64.1
	2.75	26	8.6	8.6	72.7
	3.00	31	10.2	10.2	82.9
	3.25	22	7.2	7.2	90.1
	3.50	19	6.3	6.3	96.4
	3.75	7	2.3	2.3	98.7
	4.00	2	.7	.7	99.3
	4.25	1	.3	.3	99.7
	4.50	1	.3	.3	100.0
	Total	304	100.0	100.0	

SEVERITY

		Frequency	Percent	Valid Percent	Cumulative Percent
Valid	1.00	12	3.9	3.9	3.9
	1.25	6	2.0	2.0	5.9
	1.50	13	4.3	4.3	10.2
	1.75	11	3.6	3.6	13.8
	2.00	17	5.6	5.6	19.4
	2.25	23	7.6	7.6	27.0
	2.50	25	8.2	8.2	35.2
	2.75	31	10.2	10.2	45.4
	3.00	39	12.8	12.8	58.2
	3.25	40	13.2	13.2	71.4
	3.50	25	8.2	8.2	79.6
	3.75	19	6.3	6.3	85.9
	4.00	18	5.9	5.9	91.8
	4.25	12	3.9	3.9	95.7
	4.50	4	1.3	1.3	97.0
	4.75	8	2.6	2.6	99.7
	5.00	1	.3	.3	100.0
	Total	304	100.0	100.0	

BENEFITS

		Frequency	Percent	Valid Percent	Cumulative Percent
Valid	1.00	1	.3	.3	.3
	1.67	1	.3	.3	.7
	1.83	1	.3	.3	1.0
	2.00	1	.3	.3	1.3
	2.17	1	.3	.3	1.6
	2.33	3	1.0	1.0	2.6
	2.50	6	2.0	2.0	4.6
	2.67	9	3.0	3.0	7.6
	2.83	4	1.3	1.3	8.9
	3.00	9	3.0	3.0	11.8
	3.17	17	5.6	5.6	17.4
	3.33	19	6.3	6.3	23.7
	3.50	25	8.2	8.2	31.9
	3.67	24	7.9	7.9	39.8
	3.83	42	13.8	13.8	53.6
	4.00	29	9.5	9.5	63.2
	4.17	24	7.9	7.9	71.1
	4.33	30	9.9	9.9	80.9
	4.50	23	7.6	7.6	88.5
	4.67	19	6.3	6.3	94.7
	4.83	9	3.0	3.0	97.7
	5.00	7	2.3	2.3	100.0
	Total	304	100.0	100.0	

SE

		Frequency	Percent	Valid Percent	Cumulative Percent
Valid	1.50	1	.3	.3	.3
	1.75	1	.3	.3	.7
	2.00	1	.3	.3	1.0
	2.25	4	1.3	1.3	2.3
	2.50	8	2.6	2.6	4.9
	2.75	14	4.6	4.6	9.5
	3.00	18	5.9	5.9	15.5
	3.25	19	6.3	6.3	21.7
	3.50	37	12.2	12.2	33.9
	3.75	48	15.8	15.8	49.7
	4.00	62	20.4	20.4	70.1
	4.25	33	10.9	10.9	80.9
	4.50	29	9.5	9.5	90.5
	4.75	17	5.6	5.6	96.1
	5.00	12	3.9	3.9	100.0
	Total	304	100.0	100.0	

BARRIERS

		Frequency	Percent	Valid Percent	Cumulative Percent
Valid	1.00	6	2.0	2.0	2.0
	1.20	5	1.6	1.6	3.6
	1.40	1	.3	.3	3.9
	1.60	4	1.3	1.3	5.3
	1.80	13	4.3	4.3	9.5
	2.00	12	3.9	3.9	13.5
	2.20	23	7.6	7.6	21.1
	2.40	12	3.9	3.9	25.0
	2.60	26	8.6	8.6	33.6
	2.80	21	6.9	6.9	40.5
	3.00	30	9.9	9.9	50.3
	3.20	40	13.2	13.2	63.5
	3.40	25	8.2	8.2	71.7
	3.60	30	9.9	9.9	81.6
	3.80	15	4.9	4.9	86.5
	4.00	14	4.6	4.6	91.1
	4.20	11	3.6	3.6	94.7
	4.40	9	3.0	3.0	97.7
	4.60	4	1.3	1.3	99.0
	4.80	2	.7	.7	99.7
	5.00	1	.3	.3	100.0
	Total	304	100.0	100.0	

CUES

		Frequency	Percent	Valid Percent	Cumulative Percent
Valid	1.00	8	2.6	2.6	2.6
	1.20	3	1.0	1.0	3.6
	1.40	5	1.6	1.6	5.3
	1.60	5	1.6	1.6	6.9
	1.80	8	2.6	2.6	9.5
	2.00	10	3.3	3.3	12.8
	2.20	15	4.9	4.9	17.8
	2.40	17	5.6	5.6	23.4
	2.60	22	7.2	7.2	30.6
	2.80	24	7.9	7.9	38.5
	3.00	33	10.9	10.9	49.3
	3.20	27	8.9	8.9	58.2
	3.40	35	11.5	11.5	69.7
	3.60	17	5.6	5.6	75.3
	3.80	27	8.9	8.9	84.2
	4.00	23	7.6	7.6	91.8
	4.20	6	2.0	2.0	93.8
	4.40	4	1.3	1.3	95.1
	4.60	5	1.6	1.6	96.7
	4.80	6	2.0	2.0	98.7
	5.00	4	1.3	1.3	100.0
	Total	304	100.0	100.0	

BCOVID

		Frequency	Percent	Valid Percent	Cumulative Percent
Valid	1.00	3	1.0	1.0	1.0
	1.50	2	.7	.7	1.6
	2.00	7	2.3	2.3	3.9
	2.50	40	13.2	13.2	17.1
	3.00	165	54.3	54.3	71.4
	3.50	43	14.1	14.1	85.5
	4.00	28	9.2	9.2	94.7
	4.50	10	3.3	3.3	98.0
	5.00	6	2.0	2.0	100.0
	Total	304	100.0	100.0	

VEGETABLES

		Frequency	Percent	Valid Percent	Cumulative Percent
Valid	.00	31	10.2	10.2	10.2
	.02	1	.3	.3	10.5
	.07	14	4.6	4.6	15.1
	.11	1	.3	.3	15.5
	.14	19	6.3	6.3	21.7
	.15	1	.3	.3	22.0
	.18	2	.7	.7	22.7
	.18	1	.3	.3	23.0
	.21	19	6.3	6.3	29.3
	.23	1	.3	.3	29.6
	.24	1	.3	.3	29.9
	.25	1	.3	.3	30.3
	.29	18	5.9	5.9	36.2
	.30	1	.3	.3	36.5
	.32	1	.3	.3	36.8
	.34	1	.3	.3	37.2
	.36	12	3.9	3.9	41.1
	.36	8	2.6	2.6	43.8
	.39	1	.3	.3	44.1
	.40	1	.3	.3	44.4
	.43	6	2.0	2.0	46.4

VEGETABLES

	Frequency	Percent	Valid Percent	Cumulative Percent
.43	2	.7	.7	47.0
.44	1	.3	.3	47.4
.46	1	.3	.3	47.7
.50	23	7.6	7.6	55.3
.57	16	5.3	5.3	60.5
.59	1	.3	.3	60.9
.61	1	.3	.3	61.2
.64	11	3.6	3.6	64.8
.64	1	.3	.3	65.1
.68	2	.7	.7	65.8
.71	3	1.0	1.0	66.8
.71	7	2.3	2.3	69.1
.79	3	1.0	1.0	70.1
.79	3	1.0	1.0	71.1
.82	1	.3	.3	71.4
.86	2	.7	.7	72.0
.86	3	1.0	1.0	73.0
.93	1	.3	.3	73.4
.93	2	.7	.7	74.0
1.00	14	4.6	4.6	78.6
1.04	1	.3	.3	78.9
1.07	5	1.6	1.6	80.6
1.11	2	.7	.7	81.3
1.14	9	3.0	3.0	84.2
1.21	4	1.3	1.3	85.5
1.21	1	.3	.3	85.9
1.25	1	.3	.3	86.2
1.29	4	1.3	1.3	87.5
1.29	2	.7	.7	88.2
1.32	1	.3	.3	88.5
1.36	4	1.3	1.3	89.8
1.43	4	1.3	1.3	91.1
1.50	9	3.0	3.0	94.1
1.64	3	1.0	1.0	95.1
1.64	1	.3	.3	95.4

VEGETABLES

	Frequency	Percent	Valid Percent	Cumulative Percent
1.71	1	.3	.3	95.7
1.71	2	.7	.7	96.4
1.86	1	.3	.3	96.7
2.00	2	.7	.7	97.4
2.07	2	.7	.7	98.0
2.19	1	.3	.3	98.4
2.36	1	.3	.3	98.7
2.43	1	.3	.3	99.0
2.57	2	.7	.7	99.7
6.86	1	.3	.3	100.0
Total	304	100.0	100.0	
